# Flt3 inhibition leads to decreased production of IL-6 by synovial biopsies and fibroblast-like synoviocytes by reducing NFKb activity

**DOI:** 10.1186/1479-5876-9-S2-P49

**Published:** 2011-11-23

**Authors:** M Inês Ramos, Olexandr Korchynskyi, Saida Aarrass, Paul Peter Tak, M Cristina Lebre

**Affiliations:** 1 Division of Clinical Immunology and Rheumatology, Academic Medical Center/University of Amsterdam, Amsterdam, The Netherlands

## Background

Rheumatoid arthritis (RA) is characterized by inflammation and hyperplasia of the synovial membrane which ultimately results in erosion of cartilage and bone. Fibroblast-like synoviocytes (FLS) play a critical role in this destructive process by producing inflammatory cytokines. Interleukin 6 (IL-6) is a key cytokine in the pathobiology of RA and biological therapies targeting the IL-6 receptor have shown clinical benefit. The Fms-like tyrosine kinase 3 (Flt3) is a membrane bound tyrosine kinase receptor which has a crucial role in hematopoiesis, regulating cellular differentiation, proliferation and apoptosis. Both Flt3L and its receptor are strongly expressed in RA and it has been shown that this axis exerts both pro-inflammatory and tissue destructive properties once in the joint cavity. Therefore we examined the effect(s) of a specific Flt3 inhibitor on IL-6 production by RA synovial biopsies and FLS.

## Methods

RA synovial tissue biopsies were cultured in the presence or absence of a Flt3 specific inhibitor (50-100 nM, Cep 701) for 4 days. FLS were stimulated for 6 hours with IL1b (25 pg/ml) with or without Cep 701. Cell-free tissue culture supernatants were harvested and analyzed for IL-6 and IL-8 by ELISA. To see the influence of Cep 701 in cell viability, FLS were stimulated for 24 hours or 4 days with Cep701 and MTT assay was performed. To identify the molecular mechanism underlying the effect of Cep701 on IL1b induced IL-6 production, NF-kb-dependent transcriptional activity was measured by NF-kb-dependent promoter luciferase reporter gene assay.

## Results

Cep701 efficiently suppressed spontaneous production of IL-6 by synovial biopsies and the IL1b induced IL-6 production by RA FLS. This supression was not due to increase cell death, and it is mainly due to reduction of NFKb activity.

## Conclusions

Our findings suggest that therapies targeting Flt3L/Flt3 activity may be useful in suppressing inflammation in RA.

